# Multicomponent nucleic acid enzymes as signal amplification strategy for the detection of microRNA based on fluorescence resonance energy transfer

**DOI:** 10.1007/s00604-025-07002-6

**Published:** 2025-02-25

**Authors:** Adrián Sánchez-Visedo, Patricia Alcázar-González, Luis José Royo, Ana Soldado, Francisco Javier Ferrero, José Manuel Costa-Fernández, María Teresa Fernández-Argüelles

**Affiliations:** 1 Department of Physical and Analytical Chemistry, University of Oviedo, Avenida Julián Clavería 8, 33006 Oviedo, Asturias Spain; 2 Department of Functional Biology, University of Oviedo, Avenida Julian Claveria, s/n, 33006 Oviedo, Asturias Spain; 3 Department of Electrical, Electronic, Communications and Systems Engineering, University of Oviedo, Campus Gijon, 33204 Gijón, Spain; 4 International Iberian Nanotechnology Laboratory, Av. Mestre José Veiga S/N, 4715-330 Braga, Portugal

**Keywords:** MicroRNA, FRET, MNAzyme, Signal amplification, Gold nanoparticle

## Abstract

A novel and simple methodology is introduced that allows accurate and highly sensitive detection of microRNAs (miRNAs), taking advantage of an amplification strategy based on multicomponent nucleic acid enzymes (MNAzymes), combined with a fluorescence resonance energy transfer (FRET) phenomenon. For this purpose, a fluorescent dye (FAM) has been selected as an energy donor, while gold nanoparticles (AuNPs) are employed as energy acceptors, located close to each other through hybridisation with the substrate. The research object was miR146a, which is a biomarker whose overexpression in milk is associated with inflammation in bovine mammary glands caused by bovine mastitis. The presence of a genetic target activates the MNAzyme cleavage capability, splitting the substrate into two parts. Hence, the presence of the target increases the distance between donor and acceptor, recovering the quenched fluorescence. Experimental parameters have been optimised, achieving a limit of detection (LOD) of only 2.3 fM (highly competitive as compared to other similar approaches) and a wide linear response range between 15.9 fM and 10 nM. In addition, the proposed methodology allows discriminating miR146a from other similar miRNAs differing in a single base mismatch. Detection of miR146a has been successfully carried out in spiked raw milk samples.

**Graphical Abstract:**

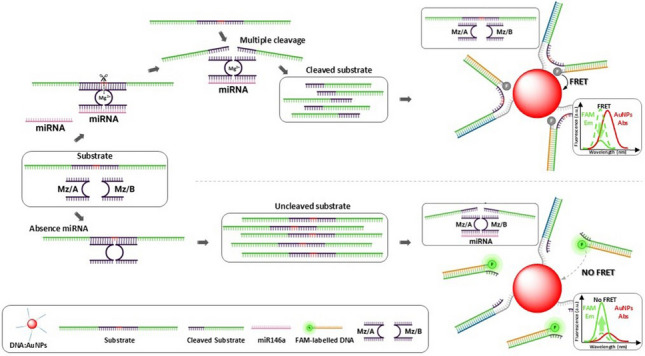

**Supplementary Information:**

The online version contains supplementary material available at 10.1007/s00604-025-07002-6.

## Introduction

MicroRNAs (miRNAs) are small, endogenous and non-coding RNA sequences (18–25 nucleotides), which are involved in numerous biological processes. Researchers have demonstrated that the downregulation and overexpression of miRNAs are early symptoms closely related to multiple diseases, including a wide variety of cancers and inflammatory diseases. Consequently, they have emerged as promising biomarkers for early clinical diagnosis. miRNAs are associated with the inflammatory response to certain infections, such as bovine mastitis, upregulating miR146a in dairy cattle [1–4]. Due to the need for monitoring bovine mastitis as a quality indicator in the milk industry, it is necessary to develop rapid and highly sensitive methodologies [5]. Despite the most common method for mastitis diagnosis is a bacteriological culture of milk, novel approaches could be based on the detection of a specific miRNA acting as a mastitis biomarker [6, 7].

Nevertheless, quantification of miRNAs is a challenging task. Their short sequence length complicates traditional quantification methods designed for longer RNA sequences. In addition, their low natural abundance and the high sequence similarity amongst miRNAs members of the same family [8] require highly sensitive and sensitive techniques for their accurate quantification. The most common methods to detect miRNAs include Northern blotting, quantitative real-time PCR (qPCR) and quantitative reverse transcription-polymerase chain reaction (qRT-PCR), small RNA sequencing or microarrays using specific probes to detect and quantify miRNAs. However, these methods are frequently characterised by insufficient sensitivity and selectivity, and they typically require large amounts of sample, complex operations and skilled personnel, making miRNA analysis challenging, time-consuming and expensive [9–11]. Therefore, it is an urgent demand to develop highly sensitive and specific methods for miRNA detection.

An alternative to improve the sensitivity is to carry out the genetic detection using nucleic acid signal amplification schemes based on protein enzymes, such as rolling circle amplification (RCA), loop-mediated isothermal amplification (LAMP) or duplex-specific nuclease signal amplification (DSNSA) [12]. Nevertheless, despite the low detection limits achieved with those strategies, activity of protein enzymes is significantly affected by components present in complex sample matrices (e.g. clinical, food or environmental samples) and their stability is highly dependent on temperature. Alternatively, signal amplification strategies based on the use of deoxyribozyme nucleic acid enzymes (DNAzymes), which elude using protein enzymes, have demonstrated to be capable of giving rise to an effective signal amplification [13]. Amongst them, multicomponent nucleic acid enzymes (MNAzymes), derived from RNA-cleaving DNAzymes, have shown a great potential in bioanalytical applications due to their catalytic efficiency and high robustness that allows working in stringent experimental conditions of temperature and pH [14, 15].

MNAzymes consist of two parts called partzymes. Each partzyme contains three different regions: the substrate arm and the sensor arm, which are partially complementary to the substrate and target, respectively; and another region that is part of the catalytic core. When the catalytic core is active, which occurs only in the presence of the target, it cleaves the substrate repeatedly, leading to a signal amplification. MNAzymes have become a highly flexible tool to detect genetic sequences due to their simple design that allows the detection of different targets by modifying the sensor arms [16]. Taking advantage of the capability of MNAzymes to break a phosphodiester bond in the substrate, it is possible to correlate the presence of a certain target by measuring a distance-dependent property. In this context, assays involving analyte-dependent agglomeration of DNA-modified AuNPs, leading to observable colour changes, provide a quick and easy detection approach. When combined with MNAzymes, these assays provide detection limits in the pM range [17, 18]. In our laboratory, we already performed visual detection of miR146a using DNA-modified gold nanoparticles (DNA-AuNPs) in combination with MNAzymes. The cleavage of the substrate, induced by the presence of the analyte (miR146a), resulted in changes in the distance between the DNA-AuNPs that could be observed with the naked eye due to the characteristic surface plasmon resonance (SPR) behaviour of AuNPs [19].

However, detection limits lower than the pM level might be required for certain applications. For such purpose, a methodology based on FRET (fluorescence resonance energy transfer) can be considered as such phenomenon is highly advantageous for achieving exceptional sensitivity in molecular detection. In this sense, AuNPs have inherent properties that make them good candidates as energy acceptors, in combination with a fluorescent donor [20, 21]. Actually, interaction between AuNPs and an organic fluorophore (FAM) has been extensively studied to develop FRET-based genetic detection assays, improving noticeably the sensitivity achieved [22]. Hence, the present work aims to combine the advantages of FRET-based fluorescence detection with the amplification capability of MNAzymes to detect very low levels of miR146a. Due to the different nature of donor and acceptor (the surface one AuNP acting as an acceptor is loaded with multiple DNA sequences that can interact with several donor molecules), special attention was paid to DNA:AuNP molar ratios, donor:acceptor ratios and experimental conditions affecting the catalytic activity of MNAzymes. Finally, usefulness of the developed FRET-based assay for miR146a quantification was evaluated in the analysis of raw milk samples.

## Materials and methods

### Materials and reagents

All nucleic acids were obtained from Integrated DNA Technologies (IDT, IA, USA) and are listed in Table 1. A thiolated DNA sequence has been employed to modify the AuNP surface. DNA substrate strand is modified with two RNA bases, represented as “rGrU”. Sodium citrate tribasic dihydrate (Na_3_C_6_H_5_O_7_·2H_2_O), hydrogen tetrachloroaurate trihydrate (HAuCl_4_·3H_2_O), Trizma® hydrochloride (NH_2_C(CH_2_OH)_3_·HCl), magnesium chloride hexahydrate (MgCl_2_·6H_2_O), potassium chloride (KCl) and Tris–Borate-EDTA buffer, 5 × concentrate, powder blend were purchased from Sigma-Aldrich (St. Louis, USA; www.sigmaaldrich.com). Thiolated metoxy polyethylene glycol (mPEG-SH_1000_) was purchased from from Laysan Bio, Inc. (Huntsville, USA). SeaKem® LE Agarose for gel electrophoresis, from Cambrex Bio Science Rockland, Inc. Rockland was employed. Raw milk samples from cows were provided by the Department of Animal Nutrition, Grassland and Forages, Regional Institute for Research and Agro-Food Development (SERIDA, Asturias, Spain). The extraction of the microRNA was made with QIAzol Lysis Reagent from QIAGEN (USA; https://www.qiagen.com) and mirVana microRNA Isolation Kit together with phosphate-buffered saline (PBS) from Thermo Fisher Scientific (USA; https://www.thermofisher.com). This microRNA Isolation Kit employs extraction columns that bind nucleic acids, which have negative charge. Therefore, the extraction of positive ions that could be present in the samples is not expected.

**Table 1:** DNA and RNA sequences employed in the present work

Name	Sequences (5′–3′)
SH-DNA	SH-AAA AAA AAA ACC TAT CGA CCA TGC T
FAM-DNA	TTT GCT GAG ATC GCG AA-FAM
Substrate	AGC ATG GTC GAT AGG TAA GGT TTC CTC rGrU CCC TGG GCA TAA ACG ACT CTA GCG C
Mz/A	ACA ACC TAT GGA ACA ACG AGA GGA AAC CTT
Mz/B	TGC CCA GGG AGG CTA GCT ATT CAG TTC TCA
miR146a	UGA GAA CUG AAU UCC AUA GGU UGU
miR146-1b	UGA GAA CUG AAU UCC AUA GGC UGU
miR146-2b	UGA GAA CUC AAU UCU AUA GGU UGU
miR146-3b	UGA GAA CUG AAU UCC AUA CCU UCU

### Synthesis of AuNPs

AuNPs were synthesised according to a previously described method [23]. Characterisation of the morphology, size and polydispersity index (PDI) of AuNPs was performed using dynamic light scattering (DLS) and transmission electron microscopy (TEM) measurements. The AuNPs synthesised and used in this work have a NP diameter of 15 ± 1 nm (*n* = 500) with a PDI of 0.02. Concentration of AuNPs was calculated by measuring the absorbance value at the maximum of the LSPR peak, using an extinction coefficient of 3.67 × 10^8^ M^−1^ cm^−1^ at *λ* = 521 nm for 15 nm AuNPs as described elsewhere [24].

### Surface functionalisation of AuNPs

Functionalisation of AuNPs was carried out using a thiolated DNA sequence as a ligand following a procedure previously described [25]. For this purpose, 20 µL of 100 nM AuNPs was placed together in an Eppendorf tube with 20 µL of 5 µM SH-DNA probe and 40 µL of a buffer containing 90 mM trisodium citrate HCl buffer (pH = 3) and 0.01% v/v Tween-20, for 30 min at room temperature. Then, 10 µL of 2 mM mPEG-SH_1000_ was added to the mixture and incubated for 30 min at 60 °C. Finally, a purification step was performed to remove the excess of both thiolated DNA and mPEG-SH_1000_ by centrifuging three times at 10,000 g for 30 min. The supernatant was discarded, and the pellet with the purified and surface-modified AuNPs was redispersed in ultrapure water with 0.01% v/v Tween-20 to a final concentration of 5.5 nM. TEM images of the AuNPs before and after bioconjugation and other data obtained from characterisation studies are included in the Electronic Supporting Information.

To estimate the optimum amount of thiolated DNA needed to achieve the maximum loading of DNA onto the AuNP surface, gel electrophoresis was run by modifying the concentration of thiolated DNA from 0 to 250 µM in the above-described bioconjugation protocol, and without adding mPEG-SH_1000_. Then, 7 µL of DNA-loaded AuNPs was mixed with 7 µL of sucrose at 40% and placed inside the wells of 1% agarose electrophoresis gel in TBE 1 × for 40 min at 100 V.

### Study of optimal ratio of DNA:AuNP and substrate-FAM concentrations for detection of miR146a

Evaluation of optimal conditions of DNA:AuNP and substrate and FAM concentrations was carried out. Samples containing 2 µL of increasing concentrations of 0 to 900 µM of the substrate and 8 µL of buffer assay, to have a final concentration in 20 µL of 300 mM of MgCl_2_, 0.1 M Trizma® HCl and 0.5 M KCl at pH = 8.3, were incubated at 50 °C for 1 h. Then, 5 µL of FAM-DNA was added to have a final concentration of 0 µM to 900 µM, and 5 µL of three different DNA:AuNP molar ratios of 50:1, 100:1 and 150:1 was incubated at 50 °C for 20 min.

### Evaluation of experimental parameters involved in MNAzyme amplification

Different concentrations of MgCl_2_ and MNAzyme subunits were evaluated. For this purpose, 1 µL of concentrations of 1, 2, 3 and 4 µM of MNAzyme subunits Mz/A and Mz/B was mixed with 2 µL of 50, 100, 150 and 300 mM of MgCl_2_. The signal was measured in the absence and presence of miR146a (1 nM), containing 300 nM substrate, 0.1 M Trizma® HCl and 0.5 M KCl at pH = 8.3. Incubation was carried out at 50 °C for 1 h. The selection of time and temperature for the assay was performed according to previous results by Mokany et al. [16] and results obtained in our laboratory [19]. After the cleavage step, 5 µL of 1.3 µM FAM-oligonucleotide and 5 µL of bioconjugated DNA:AuNPs, with 5.5 nM of AuNPs, were added to the sample and incubated at 50 °C for 20 min.

This study was performed using a 300 nM substrate and FAM-labelled oligonucleotide, 5.5 nM of AuNPs (as 50:1 molar ratio of DNA:AuNPs). The concentration of MgCl_2_ was evaluated from 5 to 30 mM, and the concentrations of Mz/A and Mz/B varied from 50 to 200 nM. The MNAzyme concentrations evaluated correspond to substrate:MNAzyme molar ratios of 6:1, 3:1, 2:1 and 1.5:1, respectively. Since each MNAzyme is capable of cleaving multiple substrate strands, lower ratios were not evaluated.

### Detection assay

The detection assay is carried out in two steps under isothermal conditions. First, the amplification step was carried out by mixing 4 µL of a standard solution containing the target (miR146a) at different concentrations (from 10 fM up to10 nM), with 1 µL of 3 µM MNAzyme (containing both subunits Mz/A and Mz/B), 2 µL of 3 µM substrate, 2 µL of MgCl_2_ 100 mM and 3 µL of buffer. Incubation was performed at 50 °C for 1 h. In the second step, 5 µL of 1.3 µM FAM-oligonucleotide and 5 µL of DNA-AuNPs (5.5 nM of AuNPs) were added to the medium and incubated for 20 min at the same temperature. Fluorescence measurements were subsequently carried out using a quartz cuvette and Varian Cary Eclipse Fluorescence Spectrophotometer. Excitation and emission wavelength were set at 495 and 520 nm, respectively.

### Selectivity assay

The selectivity of the proposed methodology has been carried out through the evaluation of the analytical response provided by sequences that differ on one (miR146-1b), two (miR146-2b) and three (miR146-3b) bases from the miR146a target sequence. The assay was carried out using 1-nM concentration of the sequences miR146-1b, miR146-2b and miR146-3b, instead of miR146a following the general procedure.

### Sample pre-treatment

Milk samples from dairy cattle were provided by the Regional Institute for Research and Agrofood Development of the Principality of Asturias (SERIDA). According to a procedure previously described [26], 50 mL of milk samples was centrifuged at 4000 g for 20 min at 4 °C to separate the different phases. Fat and serum were discarded, and the pellet containing the cells was isolated from the rest of the matrix sample. The pellet was washed twice by centrifugation cycles with PBS. Then, 2 mL of QIAzol Lysis Reagent was added to lyse the cells. Milk samples were obtained from healthy cows, so known amounts of miRNA were spiked into each lysed sample to estimate the recovery for miRNA detection using the proposed methodology. Finally, RNA extraction was performed using the mirVana kit under the experimental conditions provided by the manufacturer. This serves as isolation method for total RNA, ranging in size from kilobases down to 10-mers (ideal for miRNA, siRNA, shRNA and snRNA).

## Results and discussion

### Principle behind the amplification strategy for miRNA detection

**Fig. 1:**
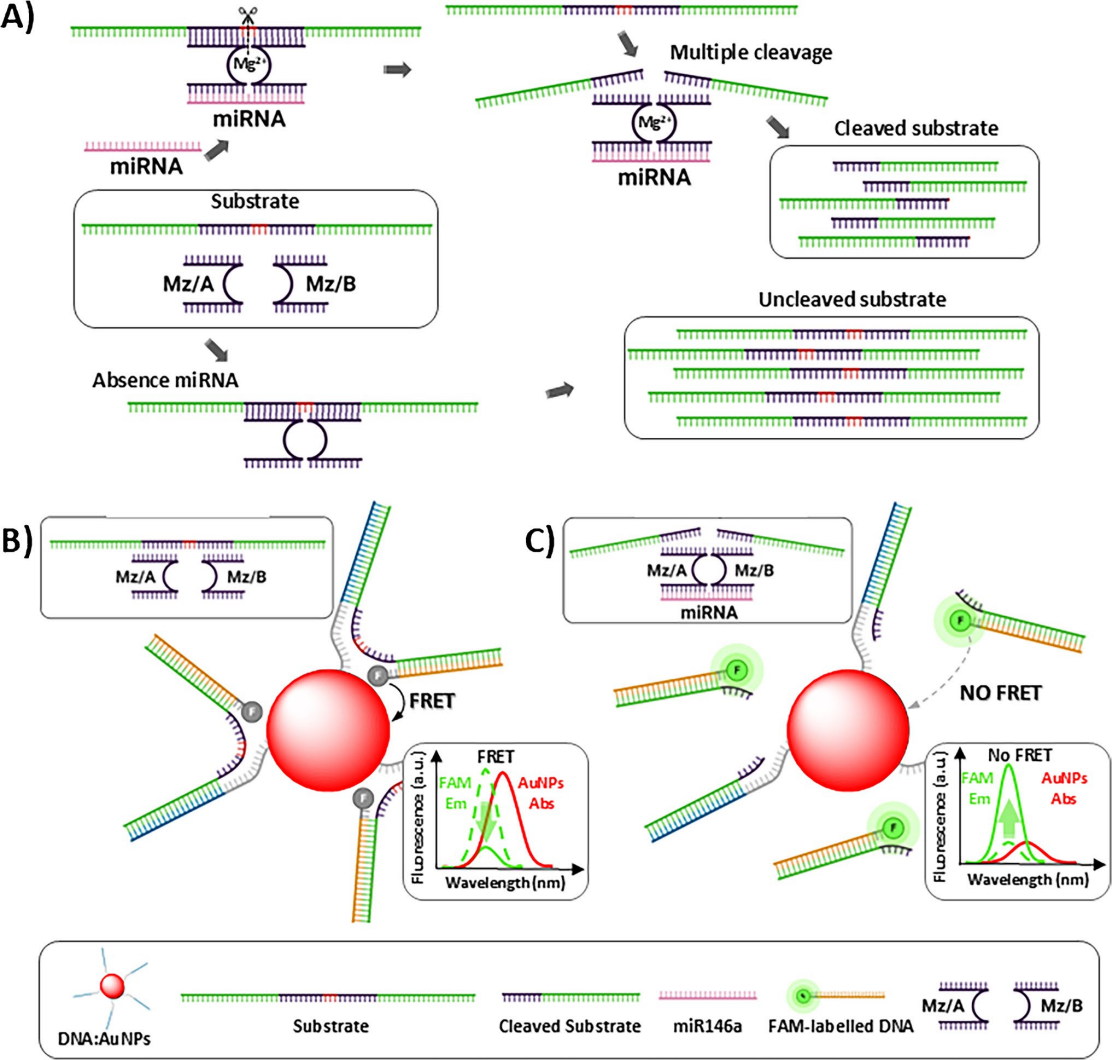
Scheme of FRET-based detection of miR146a using MNAzymes for signal amplification. **A** MNAzyme is made of two oligomers (Mz/A, Mz/B) containing two sensor arms, which are partially complementary to the target, and whose catalytic activity becomes active in a Mg^2+^ medium upon hybridisation with the target. MNAzymes also contain two substrate arms that are partially complementary to a substrate. In absence of the target, the MNAzyme is inactive, and the substrate remains intact whereas, in presence of the target, the MNAzyme becomes active, forming a structure with catalytic activity giving rise to two shorter strands. Interaction of one single target strand with an MNAzyme allows cleaving of multiple substrate strands. **B** In the absence of miR146a, the MNAzyme remains inactive, so the substrate is complete, bringing FAM (donor) molecules in close proximity to the surface of AuNPs (acceptor). FRET between the donor/acceptor pair takes place, producing a decrease of the FAM fluorescence emission. **C** In the presence of miR146a, the MNAzyme becomes active, leading to the cleavage of the substrate through the RNA bases: the donor is released far from the AuNP surface, decreasing the FRET efficiency and increasing the fluorescence signal

Figure 1 summarises the principle behind the amplification strategy for miRNA here developed, involving the use of MNAzymes in combination with DNA-functionalised AuNPs and a FRET detection approach. The detection of miRNA relies on the hybridisation of the short single-stranded RNA to complementary oligonucleotides. MNAzymes, used in this study, specifically hybridise with the target analyte. During this process, the MNAzyme catalyses the cleavage of phosphodiester bonds present in the substrate. The substrate is designed with four segments: two inner parts complementary to the MNAzyme, separated by two RNA bases to enable cleavage; and two segments complementary to oligonucleotides labelled with FAM and AuNPs, acting as donor and acceptor, respectively. Hence, in the absence of the target miRNA, the MNAzyme does not cleave the phosphodiester bonds, and the substrate remains complete, bringing in close proximity the donor and acceptor species. A high FRET efficiency occurs, and a fluorescence is quenched. Conversely, the presence of the target activates the MNAzyme, breaking the substrate into two parts, and therefore, increasing the distance between the donor and acceptor. This produces a recovery in fluorescence emission of the FAM due to a lower FRET efficiency between donor and acceptor. Hence, the presence or absence of the target can be followed through changes in the distance between the donor and the acceptor that generate changes in the fluorescence emission (see Fig. 1).

**Fig. 2:**
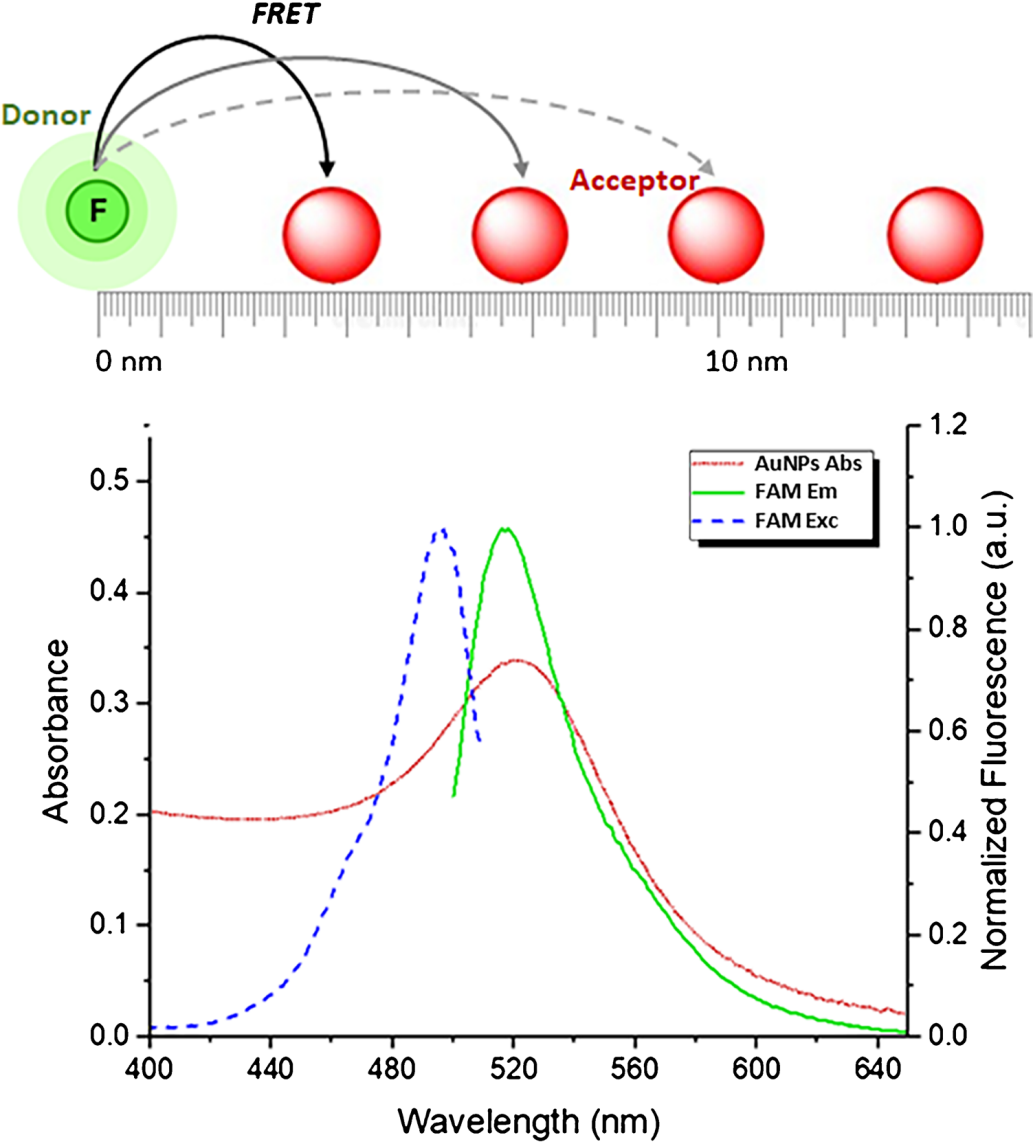
Spectral overlap between the selected donor (FAM) and the acceptor (AuNP)

The selection of a donor and acceptor was carried out in such a way that the emission spectrum of the donor overlaps the SPR peak of the AuNPs. Figure 2 shows the excitation and emission spectra of FAM (donor), which presents maximum excitation and emission wavelengths of 490 and 520 nm, respectively; and the absorption spectrum of AuNPs (acceptor). An excellent overlap between the emission of the donor and the absorption of the acceptor occurs. This allows a FRET phenomenon to take place when the donor and acceptor are in short distance.

### Optimisation of DNA:AuNP functionalisation and selection of substrate-FAM concentrations

The ligand employed for surface functionalisation of the AuNPs is a customised thiolated DNA that contains 10 adenine bases (PolyA) between the thiol group and a DNA sequence that is partially complementary to the substrate. The thiolated PolyA works as an effective anchoring block due to its preferential binding with the surface of the AuNP, favouring an upright conformation of the recognition block that facilitates hybridisation with the substrate [27]. Initially, different concentrations of thiolated DNA were assessed for functionalising the AuNP surface. The goal was to ensure both an efficient functionalisation of the AuNP surface, with a homogeneous ligand density on the AuNP surface, and an optimal performance in the recognition of the miRNA.

**Fig. 3:**
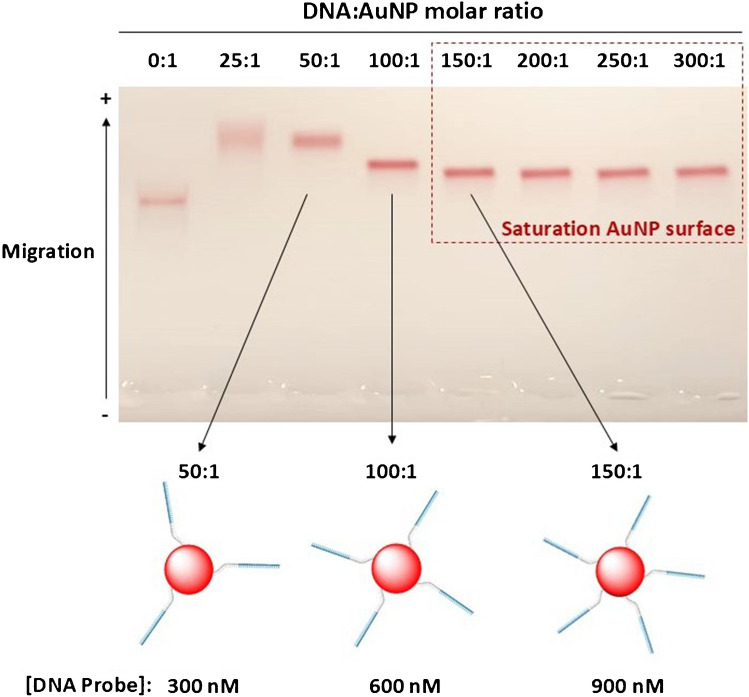
Agarose gel electrophoresis analysis of DNA:AuNP bioconjugation products prepared ensuring different DNA:AuNP molar ratios. DNA:AuNP molar ratios of 150:1 or higher give rise to AuNP bioconjugates with similar electrophoretic mobility, indicating that the AuNP surface is saturated with the DNA probe. A molar ratio of DNA:AuNPs below 50:1 leads to bioconjugates producing a broader electrophoretic band, attributed to a high variability in the AuNP surface functionalisation

For this purpose, molar ratios of DNA:AuNP from 25:1 to 250:1 were assayed for the bioconjugation reaction. Analysis of the product obtained in the bioconjugation reaction by a 1% agarose gel electrophoresis for 40 min at 100 V revealed that using a 150:1 molar ratio, the surface of the resulting AuNPs bioconjugates is saturated with DNA probes (see Fig. 3). Increasing this ratio does not give rise to AuNPs containing more DNA ligands on their surfaces. On the other hand, when the DNA:AuNP assayed ratio is below 50:1, the analysis of the bioconjugation products results in broader electrophoretic bands. This evidences that heterogeneous functionalisation of the AuNP surface has taken place in such reaction conditions.

Conversely, the ratio of DNA to AuNPs employed in the bioconjugation reaction not only impacts the colloidal stability and variability of the resulting bioconjugates, but also is a critical parameter that could influence assay performance. One AuNP acting as acceptor contains several DNA probes on its surface. Each DNA probe can interact with one substrate, and consequently, with one donor-labelled oligonucleotide. If the surface of the AuNPs contains a high density of DNA ligands, many donor molecules can be close to the AuNP, and a certain FRET efficiency will take place. Under these circumstances, the presence of a low concentration of target will break the linkage between the acceptor and few donor-labelled oligonucleotides, decreasing FRET efficiency in a low extent because many donor molecules will remain close to the acceptor. On the contrary, when the AuNPs are functionalised with a low density of DNA, few donor molecules will be close to the acceptor. The presence of a low concentration of target will break the linkage between the acceptor and the few donor moieties (FAB-labelled oligonucleotides) decorating the surface of the AuNPs, decreasing the FRET efficiency to a higher extent. Therefore, the donor:acceptor ratio is a key parameter that has to be optimised in the presence of different concentrations of substrate, which is responsible for the changes in the distance between them.

For this purpose, bioconjugates prepared using DNA:AuNP molar ratios of 50:1, 100:1 and 150:1, which correspond to 300 nM, 600 nM and 900 nM of DNA, respectively, were studied. The fluorescence signal was measured in the presence of increasing concentrations of FAM-labelled oligonucleotide and substrate (from 50 up to 900 nM), and also in the absence of substrate. The maximum fluorescence signal would be registered in the absence of substrate, whereas the presence of substrate will bring the donor and acceptor in close proximity, so the fluorescence will be minimal due to the FRET process.

In order to select the best experimental conditions, the ratio of the fluorescence measured in the absence of substrate (i.e. to mimic the fluorescence when all the substrate is broken due to the activity of the MNAzyme) to the fluorescence measured in the presence of substrate should be maximum.

For each experiment, concentrations of substrate and FAM-labelled oligonucleotide were similar, to avoid an excess of non-bonded substrate in the medium, nor an excess of FAM-labelled oligonucleotide, which would lead to an increase in the fluorescent background.

**Fig. 4:**
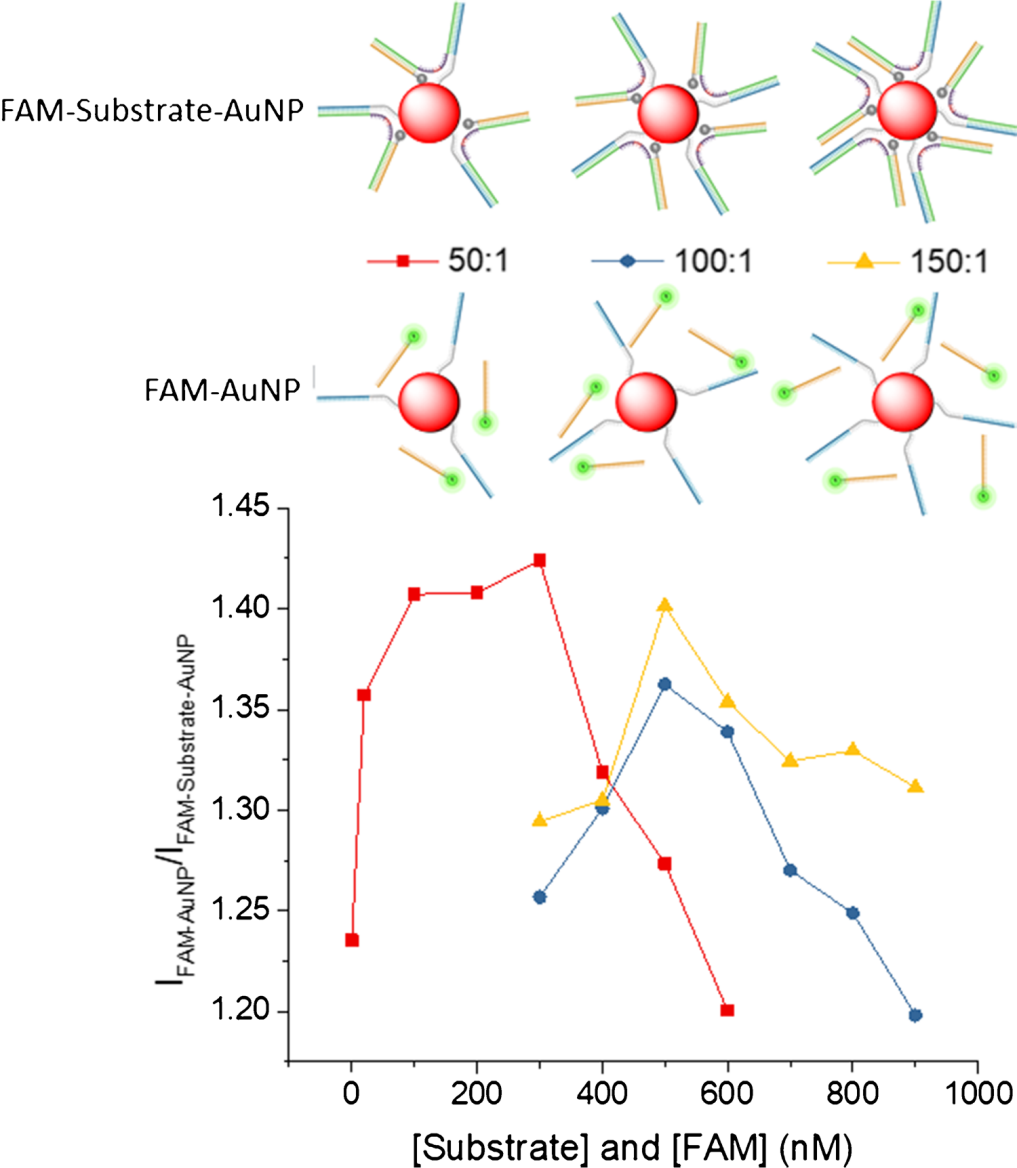
Evaluation of the fluorescence signal ratio in the presence and absence of substrate for different donor:acceptor ratios. In the absence of substrate (*I*_FAM-AuNP_), the FRET efficiency is low, and higher emission of the donor is measured. In the presence of substrate, the FRET efficiency increases due to a shorter distance between donor and acceptor, so the emission measured is lower (*I*_FAM-Substrate-AuNP_). The response to different concentrations of substrate and donor was evaluated up to 900 nM. The study was carried out with DNA:AuNP molar ratios of 50:1, 100:1 and 150 nM, respectively. The concentration of AuNPs was kept at 5.5 nM. The maximum value of the ratio of the signals measured was selected as optimum experimental conditions for further experiments

In Fig. 4, the ratio of the fluorescence of the donor/acceptor pair in the absence of substrate (*I*_FAM-AuNP_) divided by the fluorescence of the donor/acceptor pair in the presence of substrate (*F*_FAM-Substrate-AuNP_) is plotted versus increasing concentrations of substrate and donor. As can be observed, for bioconjugates prepared with a DNA:AuNP ratio of 150:1 and 100:1, the signal ratio reaches a maximum at 500 nM of substrate and FAM. Nevertheless, for the 50:1 DNA:AuNP bioconjugate, the signal ratio is maximum at substrate and donor concentrations of 300 nM. This concentration is in accordance with the concentration of thiolated DNA employed for the bioconjugation of the 50:1 DNA:AuNP bioconjugate, which was 300 nM. Consequently, further studies were carried out with the 50:1 DNA:AuNP bioconjugate using a 300 nM substrate and 300 nM of FAM-labelled oligonucleotide.

To sum up, the obtained results demonstrated that when the surface of the AuNPs is not saturated with DNA complementary to the substrate (as in the 50:1 ratio), hybridisation of the substrate to the DNA on the surface is facilitated. This promotes the proximity of the FAM molecules hybridised at the other end of the substrate, resulting in a greater fluorescence signal difference between the absence and presence of the substrate (bound to the FAM-labelled probe). In contrast, bioconjugates with higher amounts of DNA anchored to the AuNP surface showed a reduced hybridisation efficiency probably due to spatial constraints.

### Selection of experimental parameters affecting the MNAzyme amplification process

The catalytic cleavage activity of MNAzymes is affected by the presence of a divalent cation (Mg^2+^ cations have been selected in this work) and the concentration of subunits Mz/A and Mz/B that conform the MNAzyme after interaction with the target. The study was performed using a concentration of 300 nM of substrate and of FAM-labelled oligonucleotide and a 5.5 nM of DNA-AuNPs (prepared using a 50:1 molar ratio of DNA:AuNPs). MgCl_2_ concentration was evaluated from 5 to 30 mM, and concentrations of Mz/A and Mz/B varied from 50 to 200 nM. The MNAzyme concentrations evaluated correspond to substrate:MNAzyme molar ratios of 6:1, 3:1, 2:1 and 1.5:1, respectively. Taking into account that each MNAzyme is capable of cleaving multiple substrate strands, lower ratios were not evaluated.

In order to select those experimental conditions that give rise to the greatest change in the signal in the presence of the target, the ratio of the fluorescence emission in the presence of miRNA 1 nM (*F*) divided by the fluorescence emission in the absence of the target miR146a (*F*_0_) was evaluated for different concentrations of MNAzyme and Mg^2+^. The higher the *F*/*F*_0_ ratio, the greater the change in the signal in the presence of the same miRNA concentration.

**Fig. 5:**
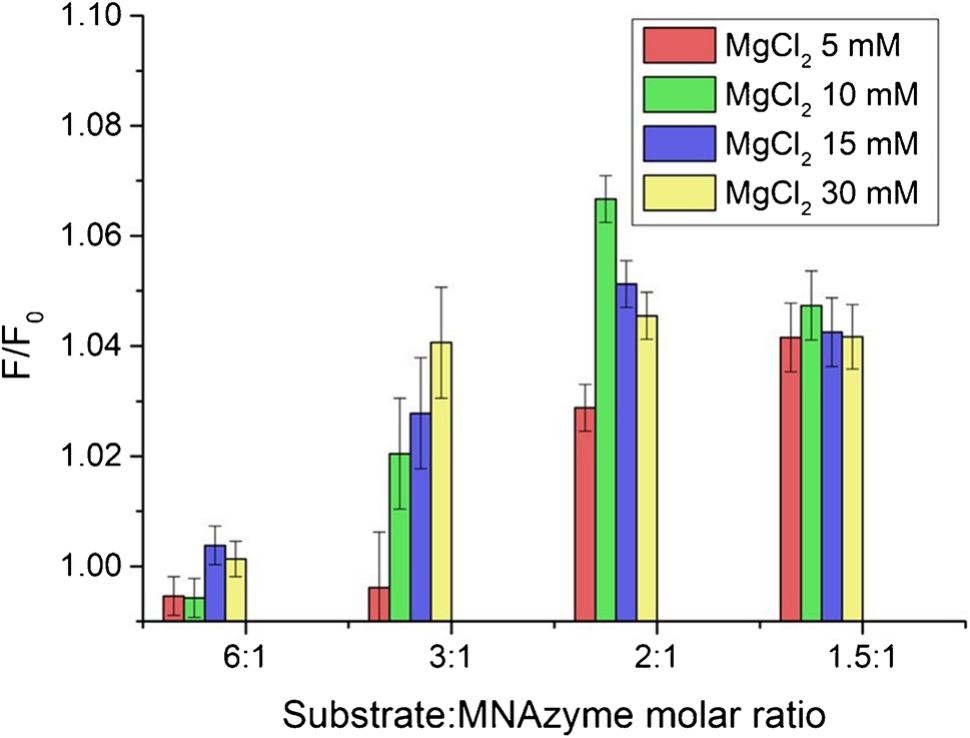
Study of the response obtained for different MNAzyme and MgCl_2_ concentrations. *F*/*F*_0_ is plotted, where *F* corresponds to the fluorescence measured in the presence of 1 nM miR146a, while *F*_0_ corresponds to the fluorescence emission without the target. Studies were carried out at constant concentrations of donor, acceptor and substrate while increasing the concentration of MNAzymes (50 nM, 100 nM, 150 nM and 200 nM) to have substrate:MNAzyme molar ratios of 6:1, 3:1, 2:1 and 1.5:1, respectively. MgCl_2_ concentrations of 30 mM, 15 mM, 10 mM and 5 mM were also evaluated

Figure 5 shows the results obtained. It is worth mentioning that the signals measured for MgCl_2_ 5 mM present poor reproducibility. This could be attributed to the lower capability of the MNAzymes to cleave the substrate at low concentrations of Mg^2+^ [17]. As can be observed, despite it could be expected that the lowest substrate:MNAzyme ratio of 1.5:1 would provide the highest *F*/*F*_0_ ratio (more MNAzyme would cleave more substrate oligonucleotides, giving rise to higher fluorescence emission), the best performance was found when ensuring a 2:1 substrate:MNAzyme molar ratio. This could be explained in terms of the probability of hybridisation, since a very high concentration of MNAzyme could minimise the hybridisation of both the target and the substrate, in the same MNAzyme pair. On the contrary, if the substrate:MNAzyme ratio is too high, the lower concentration of MNAzyme is not capable to cleave all the substrate in the same amount of time.

Regarding the substrate:MNAzyme ratio, the results obtained show a similar trend for different MgCl_2_ concentrations. Therefore, a 2:1 substrate:MNAzyme molar ratio has been selected for further experiments. Amongst the different MgCl_2_ concentrations evaluated, results obtained at 10 mM MgCl_2_ provided the highest *F*/*F*_0_ ratio and the better reproducibility of the results. This concentration was selected for further experiments.

### Analytical performance evaluation of a FRET-based assay for miR146a quantification

Detection of miR146a was carried out using the experimental conditions previously selected. First, 150 nM of Mz/A and Mz/B subunits, 300 nM of the substrate and 10 mM of MgCl_2_ were incubated for 1 h at 50 °C with aqueous standard solutions containing different miR146a concentrations ranging from 10 fM to 10 nM. During this first step, the substrate hybridises with the substrate arms of both MNAzyme subunits. The miR146a hybridises with the sensor arms of the two MNAzyme subunits, enabling the assembly of the MNAzyme catalytic core. As a result, the substrate is cleaved into two shorter oligonucleotides. In case there is no target in the medium (as in the blank), the substrate remains at its original length. In a second step, the donor and acceptor were added to the medium (300 nM of FAM-labelled oligonucleotide and 5.5 nM of DNA:AuNP bioconjugate) and incubated for 20 min at 50 °C. During this stage, if the substrate remains intact (i.e. no target has been introduced), it hybridises to both the DNA-AuNPs bioconjugate on one side of the substrate, and the FAM-labelled oligonucleotide on the opposite side of the chain. This brings the donor and acceptor into in close proximity, resulting in allow fluorescence emission. Conversely, in the presence of miR146a, the substrate undergoes cleavage. As a result, despite the donor and acceptor hybridise with both segments, now the increased distance between them leads to higher fluorescence signal.

**Fig. 6:**
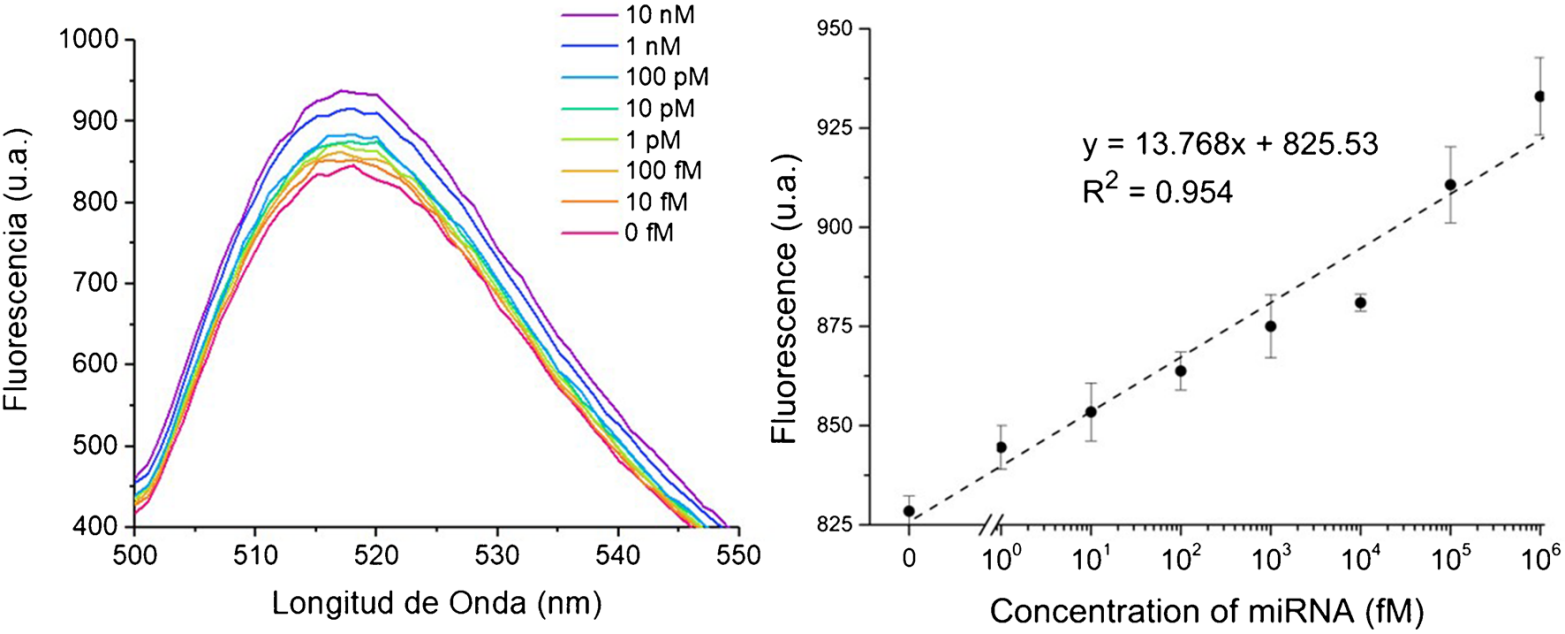
(Left) Fluorescence spectra of FAM show the increase of the signal as the concentration of the analyte is higher. (Right) Plotting the fluorescence intensity at 520 nm against miR146a concentrations of 0 fM, 10 fM, 100 fM, 1 pM, 10 pM, 100 pM, 1 nM and 10 nM. All measurements were performed in triplicate (*n* = 3)

Under the optimised experimental conditions, miR146a detection displays a concentration-dependent fluorescence signal measured at the donor’s maximum emission wavelength (520 nm). The fluorescence response measured in triplicate is linear for miR146a detection from 15.9 fM to 10 nM, as shown in Fig. 6. Moreover, under the conditions aforementioned, a limit of detection (LOD) of 2.3 fM was achieved, estimated according to the IUPAC criteria LOD = 3(*σ*/*S*), where *σ* is the standard deviation of *y*-intercepts of the calibration curve, and *S* is the slope of the calibration curve [28, 29].

**Fig. 7:**
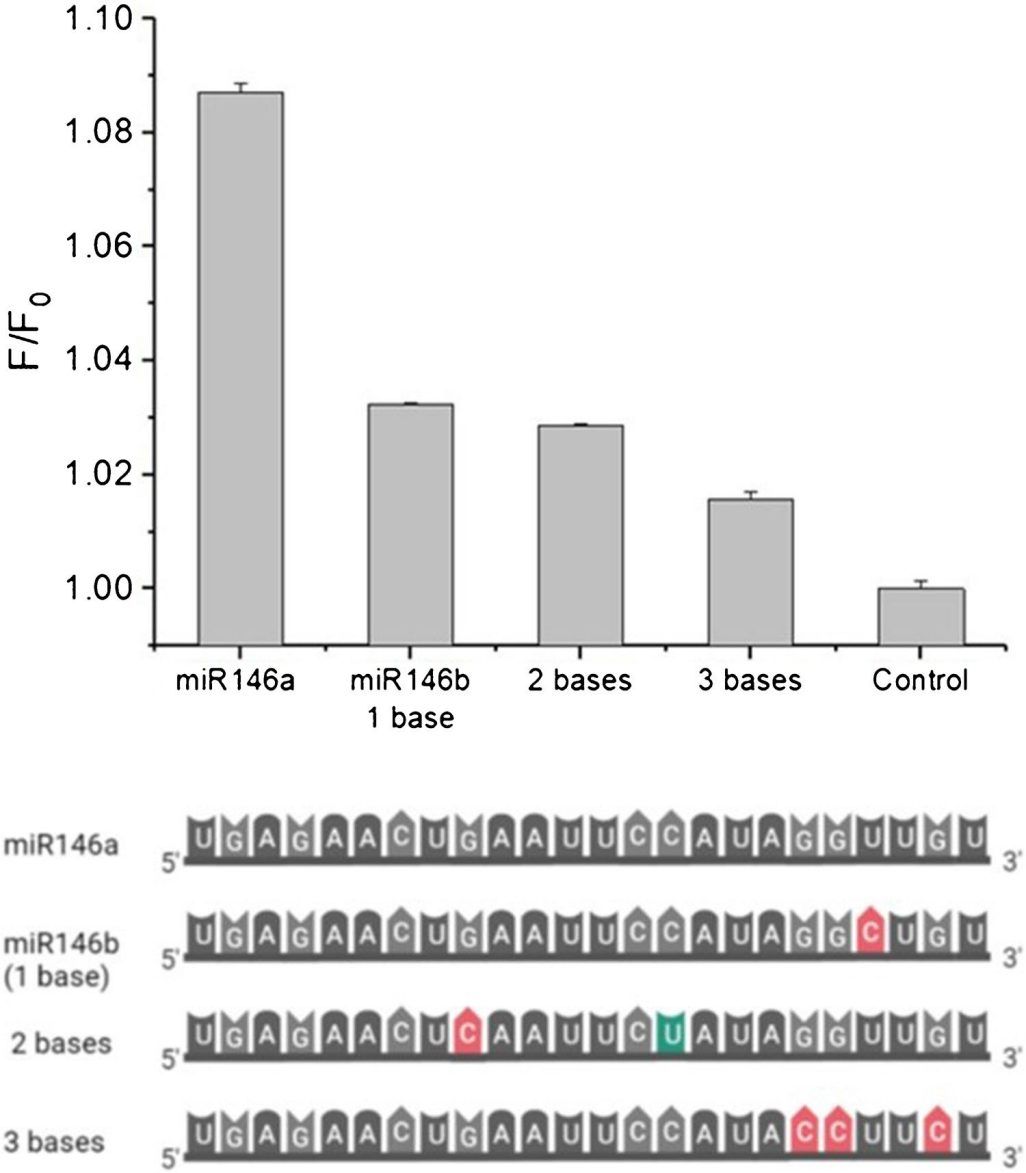
Specificity studies for miR146a detection. Bars represent the fluorescence ratio *F*/*F*_0_ obtained for different miRNA sequences: mir146a, miR146-1b, miR146-2b and miR146-3b and a control with no miRNA in the medium. Base modifications are shown in the lower part of the figure

An important challenge in miRNA analysis is to accurately discriminate between miRNA members belonging to the same family. For instance, in bovine mastitis, miR146b, which differs from the target analyte here selected miR146a in only one base, belongs to the same miR146 family. To evaluate the specificity of the here proposed methodology, the responses obtained in the presence of miRNAs that differ in one (miR146b), two (miR146a 2b-mismatch) or three bases (miR146a 3b-mismatch) from miR146a were measured. Standards of the aforementioned miRNAs were prepared at 1 nM and analysed following the general procedure. As shown in Fig. 7, the presence of 1 nM miR146a gave rise to a fluorescence emission significantly higher than the emission obtained when measuring the miRNAs with one, two or three base mismatches. These results indicate that the developed method allows the discrimination between miRNAs differing in just one base when they are at the same concentration level. However, if the sample contains higher concentration of miR146b than miR146a, the developed method is susceptible to give rise to false positive results. However, this fact does not constitute a great disadvantage, because miR146b is a miRNA that belongs to the same family than miR146a, and it is also involved in dairy cow bacterial infections.

In order to demonstrate the advantages of the method here developed compared to other approaches described for mi miRNA detection using either DNAzymes or MNAzymes, the comparative analytical features of existing methodologies are summarised in Table 1. As can be seen, the figures of merit obtained using the FRET-based methodology described in this study are favourable in terms of detection limits, even when compared to highly sensitive techniques like ICP-MS detection. Additionally, the selectivity achieved in this work is similar or surpasses that of other described approaches.

### FRET-based detection of miR146a in raw cow milk samples

The proposed methodology developed for miR146a detection has been evaluated for analysis of real samples provided by the Regional Institute for Research and Agrofood Development of the Principality of Asturias (SERIDA). For this purpose, raw milk obtained from healthy cows was spiked with known amounts of miR146a. Previous studies published in the literature have reported that miR146a in milk is mainly present in cells [26]. Therefore, separation of the different phases of milk (whey, fat and cells) was performed by centrifugation as described in the sample pre-treatment section. The cell pellet was spiked with increasing concentrations of miR146a (1 pM, 5 pM, 50 pM and 100 pM). After lysis of the cells, RNA extraction was performed using the mirVana commercial kit to purify the extracted RNA. Samples were analysed in triplicate following the standard addition method, and the results obtained are summarised in Table 2. As can be observed, the concentration measured for all samples is slightly lower than the spiked concentration. This can be attributed to the RNA extraction procedure to isolate the total RNA from the sample. Extraction of RNA, according to the literature, has a yield between 70 and 90% that has not been taken account to provide the result of measured concentration of miR146a. As shown in the table, the recovery of the detection for spiked samples is in the range within 81–94%, which is in agreement the yield of the RNA extraction kit (Table 3).

**Table 2:** Comparison of different DNAzymes and MNAzymes methods for miRNA detection

Tags	Amplification strategy	Target	Selectivity	Samples	Detection	LOD	Linear range	Ref
Carbon dots/AuNPs	DNAzymes	miR135b miR133b	45% (1 nt) (1 nM)	Bladder cancer exosome (extraction of miRNAs)	Fluorescence	*50 fM*	*50 fM to 10 nM*	[30]
Cy5/AuNPs	DNAzymes	miR21	miR155, miR141, let-7 and random RNA	Spiked in foetal bovine serum (FBS 10%)	Fluorescence	*0.13 nM*	*0.2 to 10 nM*	[9]
FAM/AuNPs	DNAzymes	miR155	1 nt (1 nM, 5 nM)	Buffer, serum and urine	Fluorescence	*50 fM*	*1 pM to 10 nM*	[10]
Magnetic beads/AuNPs	MNAzymes	miR155	1 nt (15 mM)	Pretreatment of human serum	ICP-MS	*1.15 pM*	*5 to 2000 pM*	[8]
Magnetic beads/lanthanides	MNAzymes	miR21, miR155, miR10b	1 nt (1 nM)	Spiked human serum	ICP-MS	*50 pM*	*50 to 1000 pM*	[31]
AuNPs/AuNPs	MNAzymes	miR10b	1 nt	Pretreatment of blood samples	sp-ICP-MS	*0.1 pM*	*0.1 to 25 pM*	[18]
AuNPs/AuNPs	MNAzymes	miR146a	2 nt	Pretreatment of milk samples	Visual	*250 pM*		[19]
BHQ1/FAM	MNAzymes	miRNA21, miRNA375	1 nt	Cell lysates of MCF-7, HeLa and MCF-10	Fluorescence	*0.79 pM*	*0.95 pM to 10 nM*	[32]
Streptavidin	MNAzymes-CHA	miRNA21, miRNA155	1 nt	MCF-7 total RNA	SPR	*1 pM*	*1 pM to 100 nM*	[33]
FAM/AuNPs	MNAzymes	miR146a	1 nt (1 nM)	Pretreatment of milk samples	Fluorescence	*2.3 fM*	*15.9 fM to 10 nM*	*This work*

**Table 3:** miRNA recovery concentration values (*n* = 3) after analysis of different spiked samples

Spiked (miR146a) (pM)	Measured (miR146a) (pM)	RSD (%)	Recovery (%)
1	0.8 ± 0.1	12.5	81
5	4.4 ± 0.3	6.8	88
50	44 ± 3	6.8	88
100	94 ± 5	5.3	94

## Conclusions

An ultra-sensitive methodology for miR146a detection based on a FRET detection scheme and MNAzyme signal amplification under mild isothermal conditions was developed.

Detection of miR146a can be carried out in 80 min. An extremely low LOD of 2.3 fM has been achieved, with a linear dynamic range of six orders of magnitude, enabling accurate quantification across a broad concentration spectrum. In addition, a key feature of the developed methodology is its ability to distinguish between miRNAs of the same family with only a single base mismatch. This specificity is crucial for reliable biomarker identification and disease diagnosis. The methodology was successfully evaluated in raw milk samples spiked with known amounts of miR146a. This practical application underscores the potential clinical relevance of the here developed-method for diagnosing bovine mastitis.

The innovative miRNA detection platform here developed holds promise for advancing diagnostic capabilities, especially in scenarios where high sensitivity and specificity are essential. By combining MNAzymes and FRET, we contribute to the growing field of miRNA-based diagnostics, opening new avenues for research and practical applications. By simply altering the base sequence of the genetic material used in the design of the analytical nanoplatform, it is possible to carry out the detection of other miRNA biomarkers for different diseases at ultra-low concentration levels.

## Supplementary Information

Below is the link to the electronic supplementary material.

Supplementary file1 (DOCX 2431 KB)

## Data Availability

The datasets generated and analysed during the current study are included in the manuscript and its supplementary information files. Detailed data can be found within the figures and tables presented in the manuscript. Any additional datasets that were used but are not included in the manuscript or supplementary information are available from the corresponding author upon reasonable request.
