# Supplementary material for: Multicomponent nucleic acid enzymes as signal amplification strategy for the detection of microRNA based on fluorescence resonance energy transfer
Source: Mikrochim Acta. 2025 Feb 25;192(3):186. doi: 10.1007/s00604-025-07002-6 (PMC11850480; doi:10.1007/s00604-025-07002-6)
Supplement: Supplementary file 1 — Supplementary file1 (DOCX 2431 KB) [file 604_2025_7002_MOESM1_ESM.docx]

**Electronic Supporting Information**

Multicomponent Nucleic Acid enzymes as signal amplification strategy for the detection of microRNA based on Fluorescence Resonance Energy Transfer

*Adrián Sánchez-Visedo^a,d^, Patricia Alcázar-González^a^, Luis José Royo^b^, Ana Soldado^a^, Francisco Javier Ferrero^c^, José Manuel Costa-Fernández^a^, María Teresa Fernández-Argüelles^a,^**

^a^ Department of Physical and Analytical Chemistry, University of Oviedo, Avenida Julián Clavería 8, 33006 Oviedo (Asturias), Spain

^b^ Department of Functional Biology, Genetics, University of Oviedo, Avenida Julián Clavería, s/n 33006 Oviedo (Asturias), Spain

^c^ Department of Electrical, Electronic, Computers and Systems Engineering, University of Oviedo, Campus Gijón, 33204, Gijón, Spain, University of Oviedo, Spain

^d^ International Iberian Nanotechnology Laboratory, Av. Mestre José Veiga s/n, 4715-330 Braga, Portugal

***** Corresponding author: Maria Teresa Fernandez-Argüelles Email: [fernandezteresa@uniovi.es](mailto:fernandezteresa@uniovi.es)

Phone: +34.985.103.513

ORCID codes and E-mail adress:

A. Sánchez-Visedo 0000-0002-3718-0901 adrian.visedo@inl.int

P. Alcázar-González 0009-0000-3366-061X alcazarpatricia@uniovi.es

L. J. Royo 0000-0002-5939-3000 royoluis@uniovi.es

A. Soldado 0000-0003-2483-9620 soldadoana@uniovi.es

F. J. Ferrero 0000-0003-3551-8160 ferrero@uniovi.es

J. M. Costa-Fernández 0000-0002-8671-5300 jcostafe@uniovi.es

M. T. Fernández-Argüelles 0000-0002-5191-3024 fernandezteresa@uniovi.es

**Characterization of AuNPs and DNA:AuNPs**


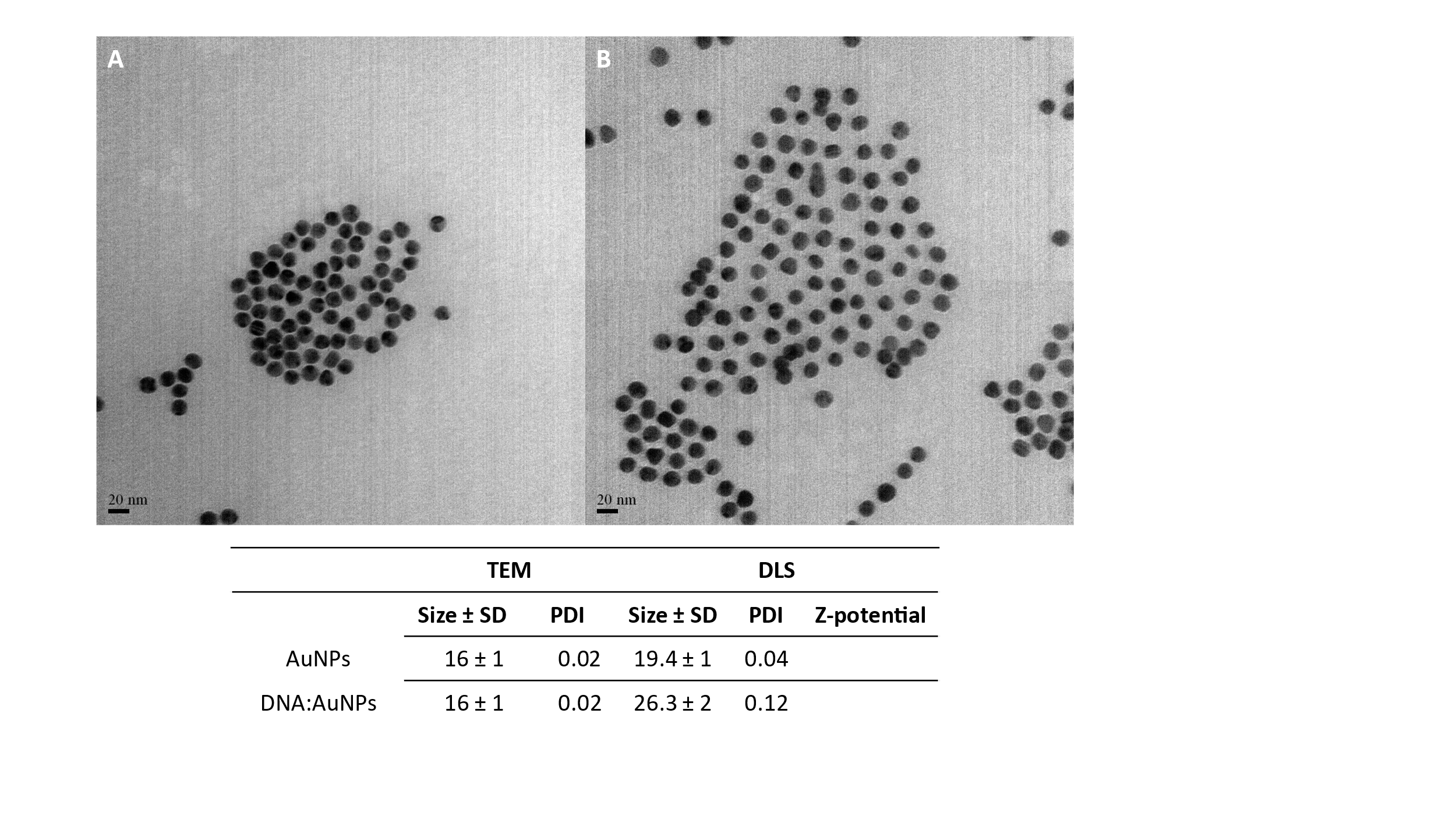


Figure 1 SI. A) AuNPs B) DNA:AuNPs

Table 1 SI. Size distribution and Z-potential values of AuNPs and DNA-covered AuNPs.

|  | **TEM** | | **DLS** | | |
| --- | --- | --- | --- | --- | --- |
|  | **Size ± SD**  **(nm)** | **PDI** | **Size ± SD**  **(nm)** | **PDI** | **Z-potential**  **(mV)** |
| **AuNPs** | 15.8 ± 1 | 0.02 | 19.4 ± 1 | 0.04 | -2.6 ± 0.2 |
| **DNA:AuNPs** | 16.1 ± 1 | 0.02 | 26.3 ± 2 | 0.12 | -7.6 ± 0.6 |


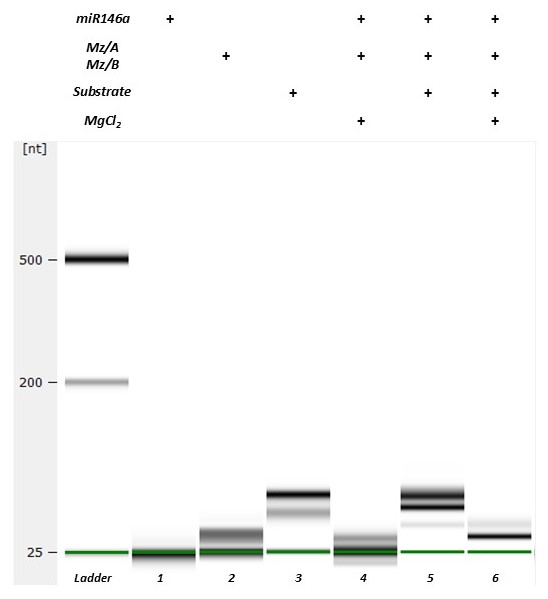


Figure 2 SI. Gel electrophoresis was performed to analyze DNA sequences involved in MNAzyme assay, using the Agilent High Sensitivity DNA Kit on the Bioanalyzer system. miRNA, MNAzyme subunits (Mz/A and Mz/B), and the substrate were evaluated in lanes 1, 2, and 3, respectively. Additionally, amplification experimental conditions (1 hour at 50 ^o^C) were applied and evaluated in lanes 4, 5, and 6. In lane 4, where miR146a and MNAzymes were tested in the presence of MgCl_2_, the bands corresponded to each specific sequence. In lane 5, the substrate was added without MgCl_2_, showing that the absence of Mg^2+^ prevents activation of the MNAzyme subunits, with the substrate sequence remaining intact in the presence of miR146a. In lane 6, the presence of MgCl_2_ and miR146a activated the MNAzyme core, resulting in substrate cleavage, and producing a band at a lower molecular size (while the band of the substrate does not appear).
